# PPT1 Reduction Contributes to Erianin-Induced Growth Inhibition in Oral Squamous Carcinoma Cells

**DOI:** 10.3389/fcell.2021.764263

**Published:** 2021-12-24

**Authors:** Qingqiong Luo, Xiaoyan Li, Guifang Gan, Meng Yang, Xu Chen, Fuxiang Chen

**Affiliations:** Department of Clinical Immunology, Ninth People’s Hospital, Shanghai Jiao Tong University School of Medicine, Shanghai, China

**Keywords:** erianin, oral squamous cell carcinoma, apoptosis, pyroptosis, protein-palmitoyl thioesterase 1

## Abstract

The anticancer properties of erianin have been recently discovered. However, the antitumor effect of erianin in oral squamous cell carcinoma (OSCC) remains unclear. In this study, we demonstrated that erianin can hamper OSCC cells growth both *in vitro* and *in vivo*. Erianin induced obvious G2/M arrest as well as apoptosis and gasdermin E (GSDME)-dependent pyroptosis in OSCC cells. Moreover, erianin increased autophagosome formation but decreased autolysosome function. Further study indicated that erianin significantly suppressed the expression of protein-palmitoyl thioesterase 1 (PPT1) and mTOR signaling. PPT1 has been reported to be a critical regulator of cancer progression by its modulation of autophagy and mTOR signaling. According to online databases, higher expression of PPT1 has been observed in OSCC tissues and is associated with poorer patient prognosis. As overexpression of PPT1 significantly reversed erianin-induced growth inhibition in OSCC cells, we identified the importance of PPT1 reduction in erianin-induced growth suppression. With the xenograft model, we confirmed the antitumor effect of erianin *in vivo*. Erianin efficiently decreased the tumor sizes, together with visibly reduced expression of PPT1 and phosphorylation of mTOR in the xenograft tumor tissues. Therefore, the present study indicated that erianin may be potentially used in OSCC therapy.

## Introduction

Oral cancer refers to malignancies that occur in the lips, gingiva, buccal mucosa, tongue, mouth floor, hard palate and retromolar tissues (Chow, 2020). Approximately 90% of oral cancers are oral squamous cell carcinomas (OSCC), and there were approximately 377,713 cases and 177,757 deaths globally in 2020 (Sung et al., 2021). Oral cancers are comparatively more frequent in South Central Asia, Eastern and Western Europe and Australia/New Zealand. The major risk factors are betel nut chewing, smoking and chronic alcohol consumption (Sung et al., 2021). Treatment of oral cancer includes surgery, chemotherapy, radiotherapy or their combination (Johnson et al., 2020). Recently, the application of immunotherapy, especially immune checkpoint inhibitors, has been introduced (Jin et al., 2020). Some patients showed great remission after this treatment regimen, however, over 50% patients relapsed. Moreover, there are many detrimental side effects caused by chemotherapy and radiotherapy, including xerostomia, loss of taste, mucositis, dermatitis, osteoradionecrosis, peripheral neuropathy and nephrotoxicity *etc.* (Romer et al., 2019; Bashir et al., 2020), which greatly affect the life quality of the patients. Therefore, there is a pressing need to find more anticancer drugs or therapies which are more efficient but with less side effects for oral cancer.

**FIGURE 1:**
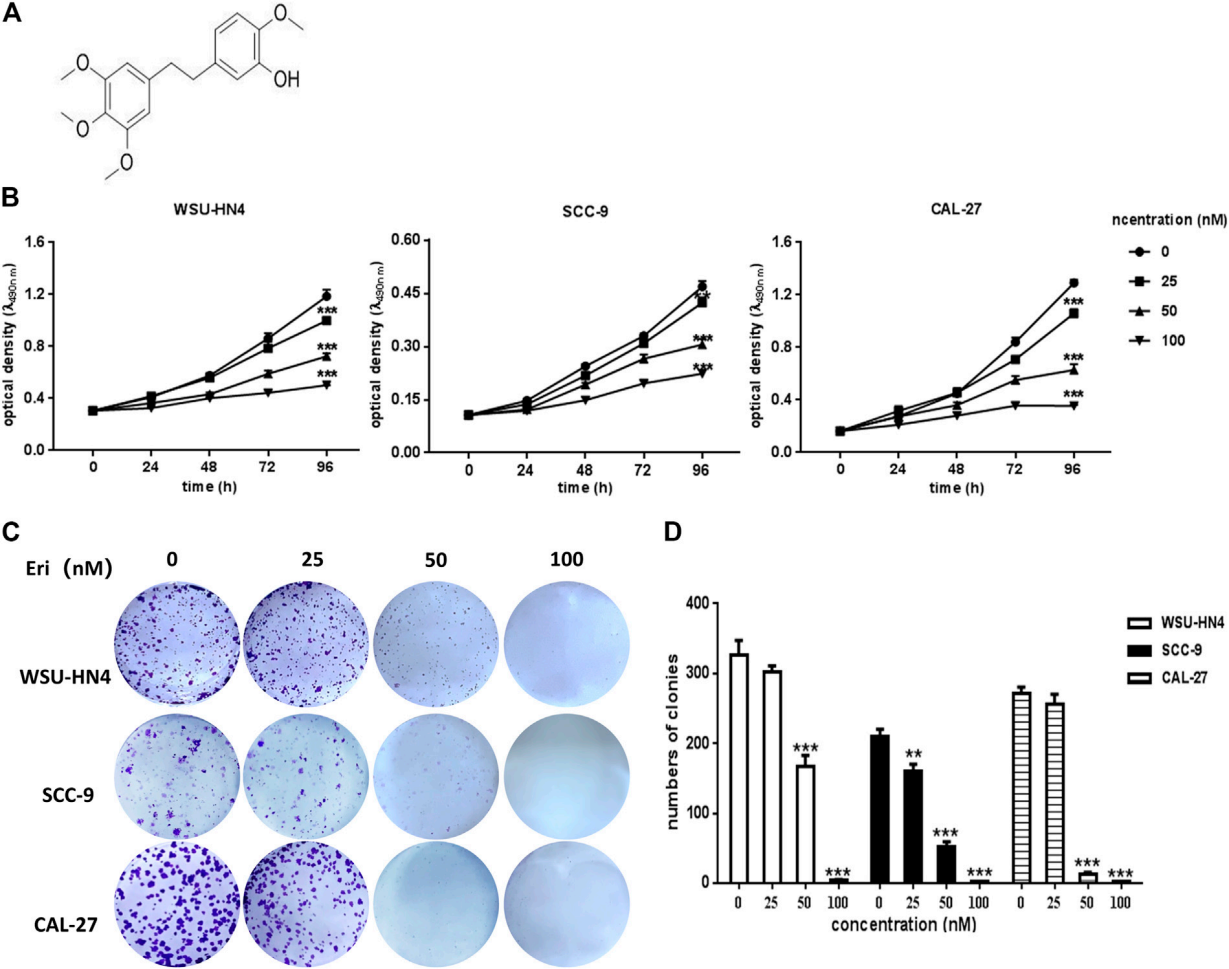
Influence of erianin on OSCC cell proliferation and colony formation. **(A)** Chemical structure of erianin. **(B)** WSU-HN4, SCC-9 and CAL-27 cells were seeded in 96-well plates. After the overnight, cells were treated with or without erianin and cell viability was analyzed by MTT assay at different time points. **(C–D)** Eight-hundred OSCC cells/well were seeded into six-well plates. After the overnight, cells were treated with or without erianin. Cell clones were stained and counted. One representative experiment out of three is shown. (***p < 0.001).

Currently, herbal products have gradually drew people’s attention for their rich natural sources and multitargeting characteristics (Kudumela et al., 2018). *Dendrobium chrysotoxum Lindl* is a traditional herb with many clinical applications, which can be traced back to the 28th century B.C. It can be used as an anti-inflammatory, analgesic, astringent and tonic agent (Wang, 2021). Erianin is a natural bibenzyl compound (Figure 1A) and it is the most noteworthy component of *Dendrobium chrysotoxum Lindl.* During the last years, the antitumor activity of erianin has been studied in a variety of human cancer cells. Wang *et al.* reported that erianin could induce G2/M-phase arrest, apoptosis and autophagy in human osteosarcoma cells (Wang et al., 2016). Dong *et al.* found that erianin deregulated mitotic regulators, caused irreparable DNA damage and also induced G2/M arrest in hepatocellular carcinoma cells (Dong et al., 2020). A study by Chen *et al.* in lung cancer cells indicated that erianin induced Ca^2+^/calmodulin (CaM)-dependent ferroptosis via ROS accumulation, lipid peroxidation and glutathione (GSH) depletion (Chen P. et al., 2020). Erianin was also found to restore the killing ability of cytotoxic T lymphocytes via suppressing programmed cell death ligand 1 (PD-L1) expression in cervical cancer cells (Yang et al., 2021). Recently, Chen *et al.* evaluated the effects of erianin on OSCC and observed increased cell cycle arrest, apoptosis and autophagy (Chen Y. T. et al., 2020). However, the underlying mechanisms and the *in vivo* anti-OSCC effects of erianin have not yet been well elucidated.

Here, we demonstrated that erianin at the nanomolar level induced obvious G2/M arrest as well as apoptosis and gasdermin E (GSDME)-dependent pyroptosis in OSCC cells. Moreover, erianin increased autophagosome formation but impaired autolysosome function. Further exploration indicated that erianin significantly reduced the expression of protein-palmitoyl thioesterase 1 (PPT1), a critical regulator of two key processes that drives cancer aggressiveness-autophagy and mammalian target of rapamycin (mTOR) signaling (Rebecca et al., 2017; Rebecca et al., 2019). PPT1 has been reported to regulate lysosomal function and is essential for the mTOR-RHEB (Ras homolog enriched in brain) interaction on the lysosomal membrane, which allows RHEB to phosphorylate and activate mTOR (Rebecca et al., 2019). As PPT1 overexpression significantly reversed erianin-induced growth inhibition in OSCC cells, we concluded that PPT1 plays an important role in erianin-induced growth suppression.

## Materials and Methods

### Animals

BALB/c nude mice (male, four-week-old) were purchased from Shanghai Laboratory Animal Center (Shanghai, China). All mice were kept in the facilities of Ninth People’s Hospital, Shanghai Jiao Tong University School of Medicine under pathogen-free conditions. The experiments were carried out according to the ethical regulations of the hospital and the Guide for the Care and Use of Laboratory Animals (The Ministry of Science and Technology of China, 2006).

### Reagents

Erianin was obtained from ChemFaces Biochemical (Wuhan, China). 3-(4,5-Dimethylthiazol-2-yl)-2,5-diphenyl tetrazolium bromide (MTT) and chloroquine were bought from Sigma-Aldrich (St. Louis, MO, USA). Ad-mCherry-p62 and Ad-mCherry-GFP-LC3B were purchased from Beyotime (Nantong, China). Apoptosis detection kit and propidium iodide (PI)/RNase staining buffer were purchased from BD Pharmingen (San Diego, CA, United States). Antibodies for Cyto C, caspase 9, cleaved caspase 3, GSDMD, CDC25C, CDC2, phospho-CDC2 (*p*-CDC2, Thr161), cyclin B1 and phospho-4EBP1 (p-4EBP1) were obtained from Cell Signaling Technology (Danvers, MA, United States); antibodies for PARP, NLRP3, 4EBP1, mTOR, phospho-mTOR (*p*-mTOR), RHEB, LC3B, beclin-1 and p62 were obtained from Proteintech Group (Wuhan, China); antibodies for cleaved-caspase 1, GSDME and PPT1 were from Abcam (Cambridge, MA, United States); and the antibody for *β*-actin was from Sigma-Aldrich. The secondary antibodies were purchased from Zhong Shan Golden Bridge Biotech Co. (Beijing, China).

### Cell Culture

WSU-HN4 cells were provided by the Key Laboratory of Stomatology, Shanghai Ninth People’s Hospital. SCC-9 (ATCC: CRL-1629) and CAL-27 (ATCC: CRL-2095) cells were purchased from Zhongyuan Biotech Co. (Beijing, China). WSU-HN4 and CAL-27 cells were cultured in Dulbecco’s modified Eagle’s medium (DMEM; Gibco-BRL, Rockville, MD, United States) supplemented with 10% fetal bovine serum (FBS; Gibco-BRL), penicillin (100 μ/mL; Beyotime, Nantong, China), and streptomycin (100 μg/ml; Beyotime). Comple culture medium for SCC-9 cells was DMEM and Ham’s nutrient mixture F12 (DMEM/F12; Gibco-BRL) medium supplemented with 400 ng/ml hydrocortisone (Sigma-Aldrich), 10% FBS, 100 μ/mL penicillin and 100 μg/ml streptomycin. All cells were kept at 37°C in a humidified 5% CO2 atmosphere.

### MTT and Clonogenic Assays

3×10^3^ cells/well were plated into 96-well plates. After the overnight, the cells were exposed to erianin at various concentrations. MTT solution (0.5 mg/ml in PBS) (Sigma) was added into each well at different time points. After an additional 4 h of incubation, the culture medium was discarded, the formazan crystals was dissolved by DMSO and the optical density was measured with a microplate reader. For the clonogenic assay, 800 cells/well were plated in 6-well plates. After the overnight, cells were treated with or without erianin. The culture medium was changed every 3 days for 2 weeks. Colonies were stained by crystal violet and were counted under a microscope.

### Cell Cycle and Apoptosis Analysis

2×10^5^ OSCC cells/well were seeded into 6-well plates. The cells were treated with or without erianin after overnight incubation. Tumor cells were collected 24 h after erianin treatment. For cell cycle analysis, the cells were fixed with cold 70% ethanol and stained for total DNA content with PI/RNase staining buffer. For apoptosis analysis, cells were double stained with FITC-conjugated annexin V and PI. Cell cycle distribution and apoptosis were analyzed by flow cytometry (Becton Dickinson, San Jose, CA, United States).

### Lactate Dehydrogenase Release and IL-1β Determination

After erianin treatment, the cell culture medium was collected. LDH activity was then determined by using an LDH detection kit. The optical density was detected at 450 nm using a microplate reader. For the determination of IL-1β, we used an Immulite 1,000 chemiluminescence instrument and matching reagents (Siemens, Germany). Six multiple wells were set for each concentration, and the experiment was repeated three times.

### Ad-mCherry-GFP-LC3B and Ad-mCherry-p62 Infection

Ad-mCherry-GFP-LC3B and Ad-mCherry-p62 were employed to monitor autophagic flux. In brief, OSCC cells (2×10^4^ cells/well) were added into a 12-well plate. At the second day, the cells were infected with Ad-mCherry-GFP-LC3B (multiplicity of infection = 50) or Ad-mCherry-p62 (multiplicity of infection = 50) for 24 h. Then the culture medium was changed with fresh medium and the cells were cultured for another 24 h. The cells were treated with erianin afterwards. Finally, the cells were washed with PBS for five times and observed with a fluorescence microscope (Olympus, Tokyo, Japan). For Ad-mCherry-GFP-LC3B transfection, both fluorophores fluoresced at neutral pH, indicating the presence of autophagosomes (yellow). When an autophagosome fuses to a lysosome during autolysosome maturation, the lower pH in the lysosome quenches the GFP signal, which shows red fluorescence (autolysosome). For cells transfected with Ad-mCherry-p62, mCherry-p62 usually diffuses within the cell under normal conditions. The formation of autophagosomes typically increases the puncta or speckles of mCherry-p62, while autolysosome maturation leads to p62 degradation and reduces the number of red speckles. When autolysosome maturation is inhibited, the red spots or patches increase in size and number.

### Proximity Ligation Assay

OSCC cells were seeded on glass cover slips and treated with erianin as described previously. After treatment for 24 h, the cells were fixed by 4% formaldehyde and permeabilized by 0.2% Triton X-100. Then the cells were blocked with 1% BSA. Later the cells were incubated with the mixture of rabbit anti-RHEB polyclonal antibody and mouse anti-mTOR monoclonal antibody at 4°C overnight. After washing for three times, the cells were incubated with the secondary antibodies conjugated with the oligonucleotides of anti-rabbit PLUS (Sigma-Aldrich, DUO92003) and anti-rabbit MINUS (Sigma-Aldrich, DUO92005), followed by the addition of ligase-ligase solution. The cells were finally subjected to microscopic analysis after the addition of amplification-polymerase solution.

### Western Blot Analysis

OSCC cells were lysed and total protein were collected and denatured as described previously. Then, the samples were electrophoretically separated on SDS-PAGE gels (EpiZyme, Shanghai, China) and transferred to a PVDF membrane (Bio-Rad, Hercules, CA, United States). After blocking with 5% nonfat milk in TBS/Tween 20 (TBS/T), the membrane was incubated with primary antibodies overnight. Immunoblots were analyzed by the Odyssey Infrared Imaging system (LI-COR Biosciences, Lincoln, NE, United States).

### PPT1 Overexpression

Commercially available PPT1 overexpression lentivirus particles were purchased from GeneChem Biotech Co. (Shanghai, China). The lentivirus-based expression plasmid Ubi-MCS-3FLAG-CBh-gcGFP-IRES-puromycin constructs was used to carry to PPT1 sequences. The primers for PPT1 were 5′-CTC​TAG​AGG​ATC​CCG​CCA​CCA​TGG​CGT​CGC​CCG​GCT​GCC​TG-3′ (forward) and 5′-AGT​CCA​TAC​CTC​CAA​GGA​ATG​GTA​TGA​TGT​GGG​CAT​A-3′ (reverse). Cells were transfected with PPT1 and vector (multiplicity of infection = 10) lentivirus-based plasmid constructs diluted with enhanced infection solution (GeneChem). Cells were collected for further processing 72 hours after transfection.

### *In Vivo* Antitumor Activity

5×10^6^ WSU-HN4 cells were subcutaneously injected into the backs of nude mice next to the forelimb and permitted to grow until the tumors were palpable. Then, the mice were randomly grouped. For the treatment group, mice were injected with 25 mg/kg or 50 mg/kg erianin intraperitoneally (i.p.) every other day for a total of 15 days. Mice in the control group received vehicle injection accordingly. With a vernier caliper, tumors were measured every 3 days. And tumor volumes were calculated as followed: tumor volume (mm^3^) = a×b^2^×0.52. In the formula, a is the longest diameter and b is the shortest diameter. Body weight of all the mice was also recorded each time. When the experiment came to the end, blood samples of the mice were obtained. Tumors were weighed after being separated, fixed with 4% paraformaldehyde and finally embedded in paraffin. Serum alanine aminotransferase (ALT) and aspartate aminotransferase (AST) activities AST activities were measured on the Dimension RXL MAX Chemistry Analyzer (Dade Behring Inc., Newark, United States).

### Immunohistochemical Staining

To determine the expression of PPT1 and *p*-mTOR in tumor tissues of nude mice, IHC staining was performed. Briefly, five-μm-thick paraffin tumor sections were deparaffinized and rehydrated. After heat-induced antigen retrieval and blocking with normal goat serum, endogenous peroxidase activity was removed. Primary antibodies were added onto the sections and were allowed to incubate overnight at 4°C. HRP-conjugated secondary antibody was incubated at room temperature the next day. Sections were finally developed in DAB solution and counterstained with hematoxylin.

### Statistical Analysis

All experiments were repeated at least three times, and representative data are shown. Student’s *t*-test was used to calculate the significance of the differences, and data was reported as the means ± SD. The value of *p* < 0.05 was considered statistically significant.

## Results

### Erianin Reduced the Viability and Proliferation of OSCC Cells *In vitro*

To confirm the antitumor potential of erianin in OSCC cells, WSU-HN4, SCC-9 and CAL-27 were employed for erianin treatment, and their viability was evaluated by the MTT assay. Generally, the viability of all three OSCC cells was reduced by erianin treatment, which exhibited negative correlation with the dose of erianin (Figure 1B). The IC50 was approximately 50 nM. The colony formation assay was adopted to determine whether erianin inhibited the growth of OSCC cells *in vitro* (Figure 1C). As expected, erianin significantly reduced the colony numbers of WSU-HN4, SCC-9 and CAL-27 cells in a dose-dependent manner (Figure 1D). Therefore, erianin surppressed the proliferation of OSCC cells *in vitro*.

### Erianin Blocked Cell Cycle Progression at the G2/M Phase in OSCC Cells

**FIGURE 2:**
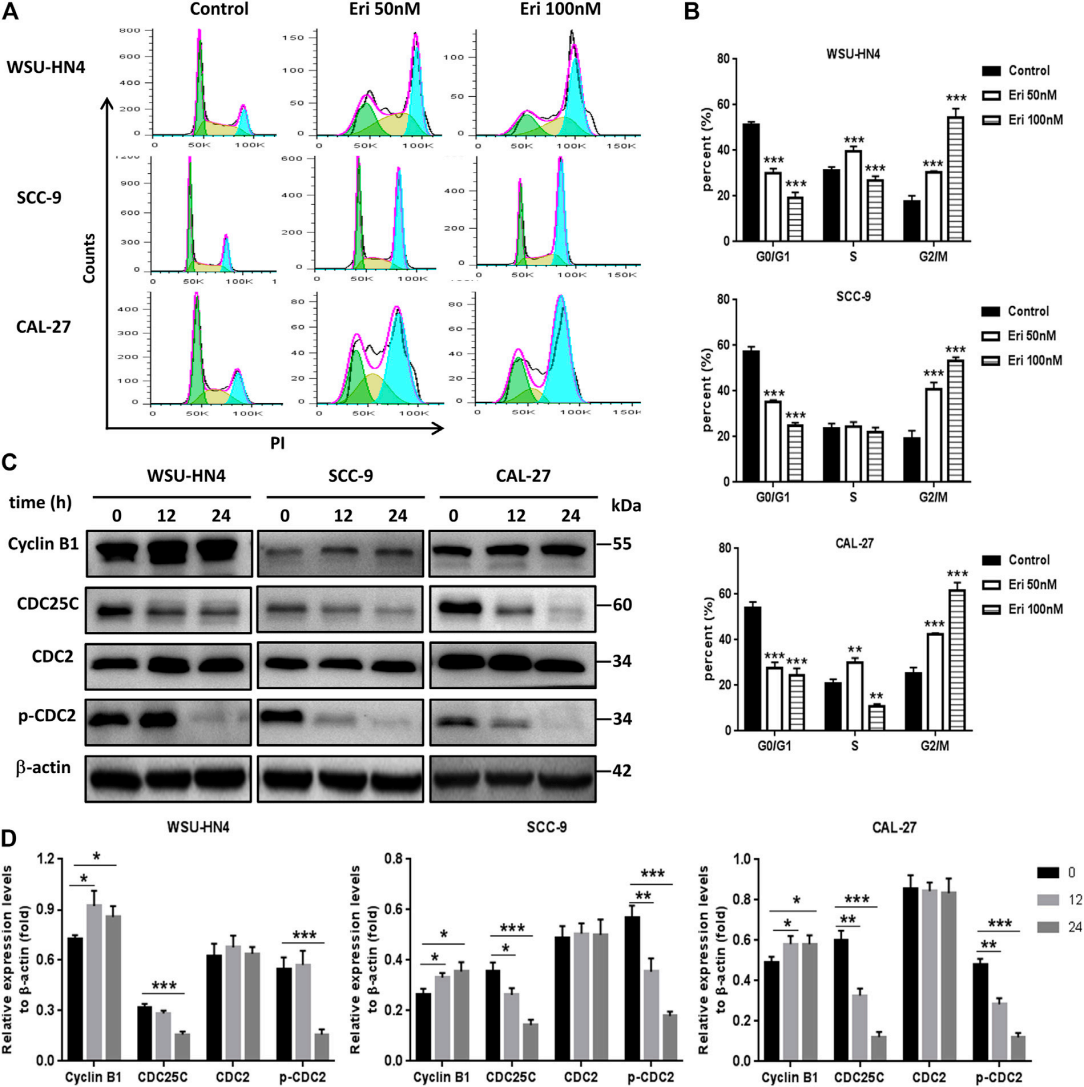
Erianin induces G2/M cell cycle arrest. Tumor cells were seeded into six-well plates. The culture medium was removed the next day and replaced by fresh medium without (control) or with 50 or 100 nM erianin for an additional 24 h of incubation. **(A)** Flow cytometric analysis of the OSCC cells. **(B)** Tumor cells at different cell cycle phases shown by histograms. **(C)** Immunoblotting assays of related cell cycle regulatory proteins. **(D)** Gray scale analysis of the western blot bands. One representative experiment out of three is shown. (***p < 0.001, versus the control group).

To determine the manner in which erianin inhibited OSCC growth, the cell cycle distribution of OSCC cells with or without erianin treatment was analyzed by flow cytometry. The results indicated that erianin increased cell numbers at G2/M phase, accompanied by reduced cell numbers at G0/G1 and/or S phases in the OSCC cell lines (Figures 2A,B). These data suggested that erianin cuased G2/M-phase arrest in OSCC cells.

In regulating the progression of the cell cycle, cyclins and CDKs play important roles (Grana and Reddy, 1995). The progression of G2/M transition and mitotic phase is under the regulation of cyclin B1. Cyclin B1 increases in the G2 phase, peaks in metaphase, and is degraded during anaphase. By coupling with cyclin B1, Cdc2 promoted maturation promoting factor (MPF) production, thus regulate the G2 phase transition to M phase. The cyclin B1/Cdc2 complex is regulated by Cdc25c *via* removing the inhibitory phosphorylation of Thr14/Tyr15 residues on Cdc2 and also by MO1518 *via* activating the phosphorylation of Cdc2 at the Thr161 residue. Our results indicated that cyclin B1 expression was upregulated, while Cdc25c and *p*-Cdc2 (Thr161) levels were downregulated (Figures 2C,D). Therefore, erianin modulated the expression of G2/M transition-related proteins, thus blocking cell cycle progression.

### Erianin Induced Apoptosis and Pyroptosis in OSCC Cells

**FIGURE 3:**
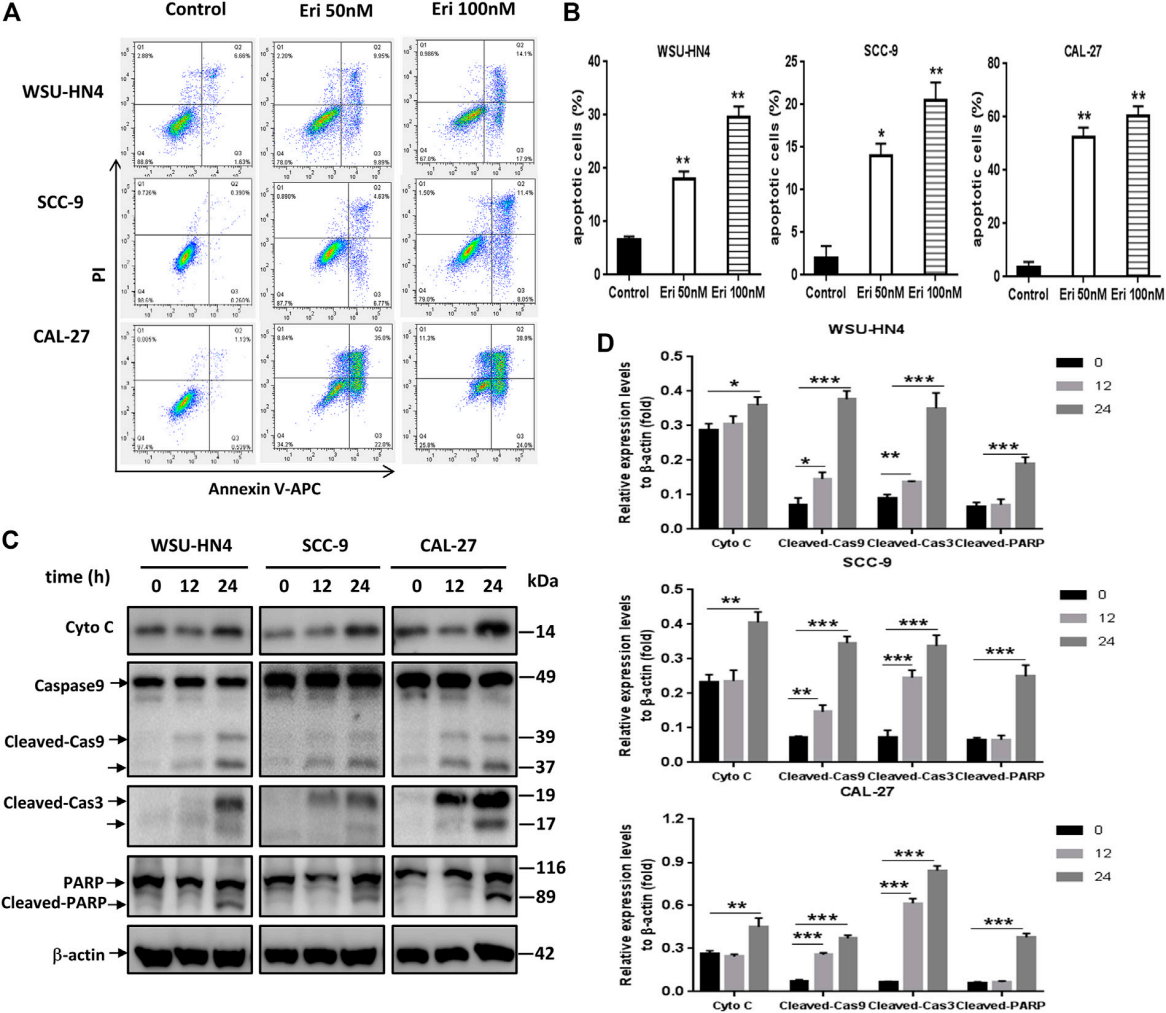
Erianin triggers apoptosis of OSCC cells. Tumor cells were seeded into six-well plates. The culture medium was removed the next day and replaced by fresh medium without (control) or with 50 or 100 nM erianin for an additional 24 h of incubation.**(A)** Flow cytometric analysis of the OSCC cells. **(B)** Apoptotic cells shown by histograms. **(C)** Western blot analysis of apoptosis-related proteins in OSCC cells. **(D)** Gray scale analysis of the western blot bands. One representative experiment out of three is shown. (*p < 0.05, **p < 0.01, ***p < 0.001).

Next, erianin-induced apoptosis in OSCC cells was investigated. OSCC cells were treated with erianin for 24 h, and apoptosis was examined through analysis of annexin V-FITC- and PI-stained cells. With erainin treatment, annexin V-FITC-positive cells increased significantly in all three OSCC cell lines (Figures 3A,B).The expression levels of apoptosis-related proteins, including Cyto C, caspase 9, cleaved caspase 3 and PARP, in erianin-treated OSCC cells were examined by western blot assay. We found that erianin increased Cyto C expression, induced the activation of caspase 9 and caspase 3, and increased the cleavage of PARP, which is the mediator of apoptosis (Figures 3C,D). These data indicated that apoptosis was involved in the decrease of OSCC cell viability induced by erianin.

### Erianin Induced GSDME-Mediated Pyroptosis in OSCC Cells

**FIGURE 4:**
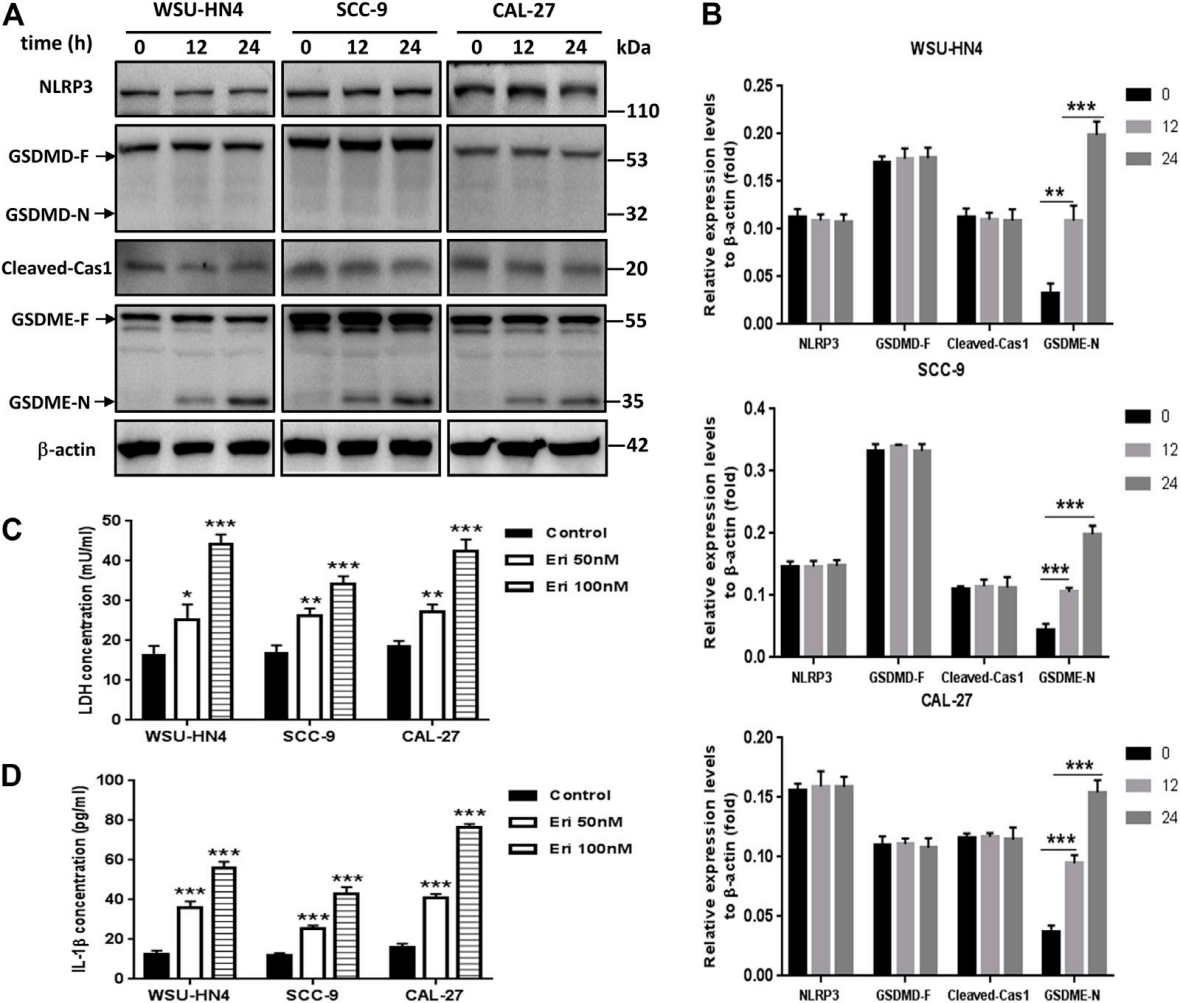
Erianin induced pyroptosis of OSCC cells. Tumor cells were seeded into six-well plates. The culture medium was removed the next day and replaced by fresh medium without (control) or with 50 or 100 nM erianin for an additional 24 h of incubation.**(A)** Western blot analysis of pyroptosis-related proteins in OSCC cells. **(B)** Gray scale analysis of the western blot bands. **(C)** LDH and **(D)** IL-1β concentrations in the culture supernatant. **(D)** One representative experiment out of three is shown. (*p < 0.05, **p < 0.01, ***p < 0.001).

Recently, pyroptosis has recently attracted great attention owing to its contributory role in cancer progression or anti-cancer therapy. Pyroptosis is a kind of lytic programmed cell death that is mainly mediated by the gasdermin family, which usually share a pore-forming domain (Liu et al., 2021). To figure out whether pyroptosis participated in erianin-induced decrease of cell viability, we first checked the expression of GSDMD, which is the most well characterized executioner of pyroptosis (Liu et al., 2021). We also examined the expression of cleaved-caspase 1, the key regulator of GSDMD cleavage. As NLRP3 inflammasome activation usually leads to the autocleavage of caspase-1 (Xue et al., 2019) and has pro-tumor growth effect in OSCC cells (Huang et al., 2017; Wang et al., 2018), we further detected the expression of NLRP3. Results showed that erianin didn’t induce the cleavage of GSDMD, neither affect the expression of NLRP3 nor cleaved-caspase 1. However, erianin treatment significantly increased the GSDME-N fragments in OSCC cells (Figures 4A,B). GSDME has been identified to be cleaved by activated caspase-3 to generate GSDME-N fragment, which executes pyroptosis by forming pores in the plasma membrane (Chen et al., 2021; Wang et al., 2021). Besides, there was markedly increased LDH and IL-1β in the culture medium of erianin-treated OSCC cells (Figures 4C,D). Therefore, we concluded that erianin induced GSDME but not GSDMD dependent pyroptosis.

### Erianin Impaired Autophagic Flux in OSCC Cells

**FIGURE 5:**
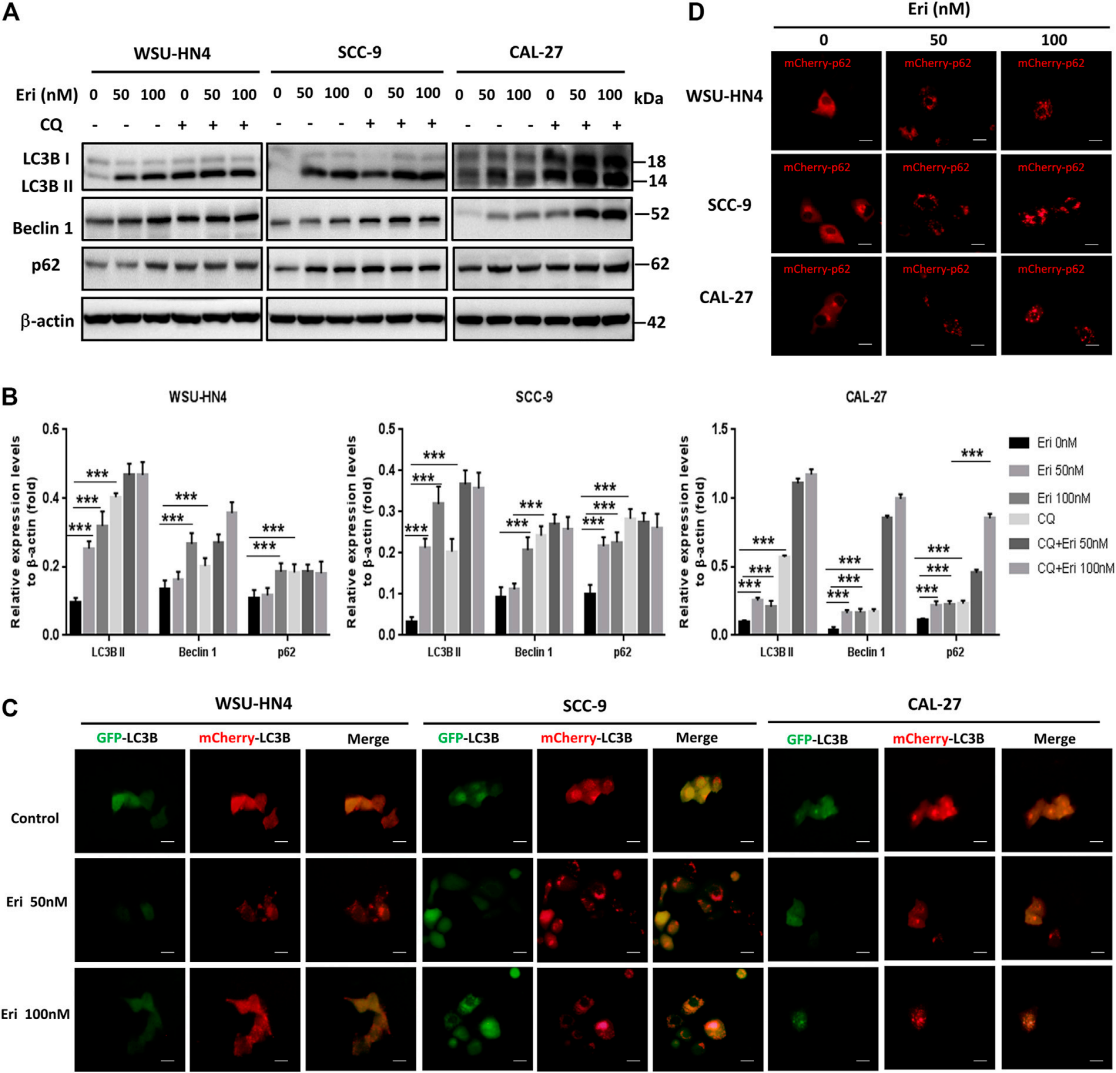
Erianin impairs autophagic flux in OSCC cells. **(A)** Tumor cells were seeded into 6-well plate and were treated as described previously. The expression of autophagy-related proteins in OSCC cells were measured. **(B)** Gray scale analysis of the western blot bands. **(C)** Adenoviruses with mCherry-GFP-LC3B plasmid and **(D)** mCherry-p62 plasmid were transfected into OSCC cells and treated as described in the Materials and Methods. Cells were observed by fluorescence microscopy. (Scale bars, 20 μm).

Autophagy has been reported to modulate cancer cell survival or death during cancer progression and therapy (Niklaus et al., 2021; White et al., 2021). To make it clear whether erianin triggered autophagy in OSCC cells, we included the autophagy inhibitor chloroquine (CQ) (Bonam et al., 2020; Klionsky et al., 2021) and performed a series of experiments. By western blot assay, we found that CQ treatment induced the accumulation of LC3B II, Beclin 1 and p62 in OSCC cells. CQ was identified to impede autophagy by inhibiting the degradation of autophagosomes in autolysosomes (Bonam et al., 2020; Klionsky et al., 2021). Similar expression patterns of these proteins were found in OSCC cells treated with erianin (Figures 5A,B). Thus, we speculated that erianin also impaired autophagic flux in OSCC cells at late phase of autophagy.

To make this result more intuitive, adenoviruses with mCherry-GFP-LC3B and mCherry-p62 were transfected into OSCC cells, and the cells were observed by fluorescence microscopy. Under normal condition, mCherry-GFP-LC3B was mainly dispersed in the cytoplasm and due to the combined effects of mCherry and GFP, the control cells usually exhibited dispersed yellow fluorescence. Yellow puncta in the cytoplasm generally indicate the formation of autophagosomes. When the autophagosomes and lysosomes fuse (autolysosome formation), GFP fluorescence is gradually quenched and LC3B is displayed in the form of red spots. Interestingly, we found increased red spots in OSCC cells treated with 50 nM erianin and more yellow puncta in 100 nM erianin-treated cells, indicating the autolysosome formation impairment (Figure 5C). P62 is usually degraded in autolysosomes during autophagy. With mCherry-p62 transfection, we found an increase in the size and number of red spots in OSCC cells treated with erianin for 24 h, which suggested inhibition of autolysosomes (Figure 5D). Therefore, these data demonstrated that erianin impeded autophagic flux.

### Erianin Suppressed PPT1 Expression and mTOR Signaling in OSCC Cells

**FIGURE 6:**
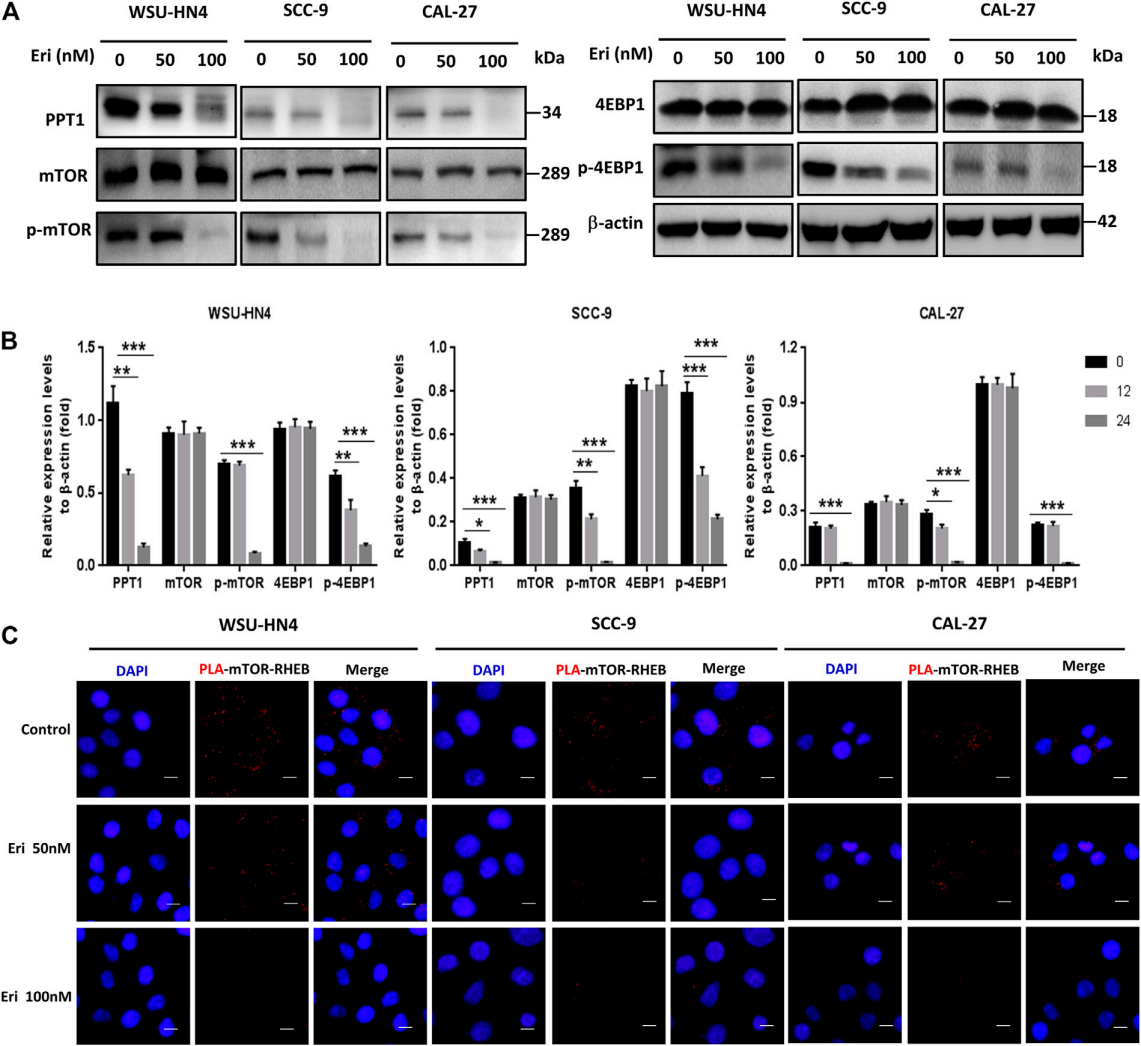
Erianin decreases PPT1 expression and affects mTOR signaling in OSCC cells. Tumor cells were plated into 6-well or 12-well plates and treated as described. **(A–B)** The expression of PPT1 and mTOR signaling-related proteins in OSCC cells with or without erianin treatment were determined. **(C)** The PLA assay was performed to determine the interaction between mTOR and RHEB. (Scale bars, 10 μm).

We next went further to determine how erianin impaired autolysosome function in OSCC cells. As PPT1 is reported to be a crucial regulator of lysosomal function that modulates autophagy and mTOR signaling (Rebecca et al., 2017), we first checked PPT1 expression in OSCC cells. Significantly decreased PPT1 expression was found in erianin-treated OSCC cells (Figures 6A,B). Moreover, the phosphorylation of mTOR and downstream 4EBP1 (eukaryotic initiating factor 4E binding protein 1) was notably inhibited by erianin, indicating the impairment of mTOR signaling (Figures 6A,B). To make this conclusion more intuitive, a proximity ligation assay was performed. As shown in Figure 6C, many red dots were observed in control OSCC cells, suggesting the vibrant interaction between mTOR and RHEB and activation of the mTOR pathway. However, erianin reduced this interaction, resulting in a marked decrease in the number of red dots in OSCC cells. The less mTOR interacts with RHEB, the less mTOR is phosphorylated. Therefore, these data implied that PPT1 reduction and subsequent mTOR signaling impairment may be involved in erianin-induced growth inhibition in OSCC cells.

### PPT1 Reduction Contributed to Erianin-Induced Growth Inhibition in OSCC Cells

**FIGURE 7:**
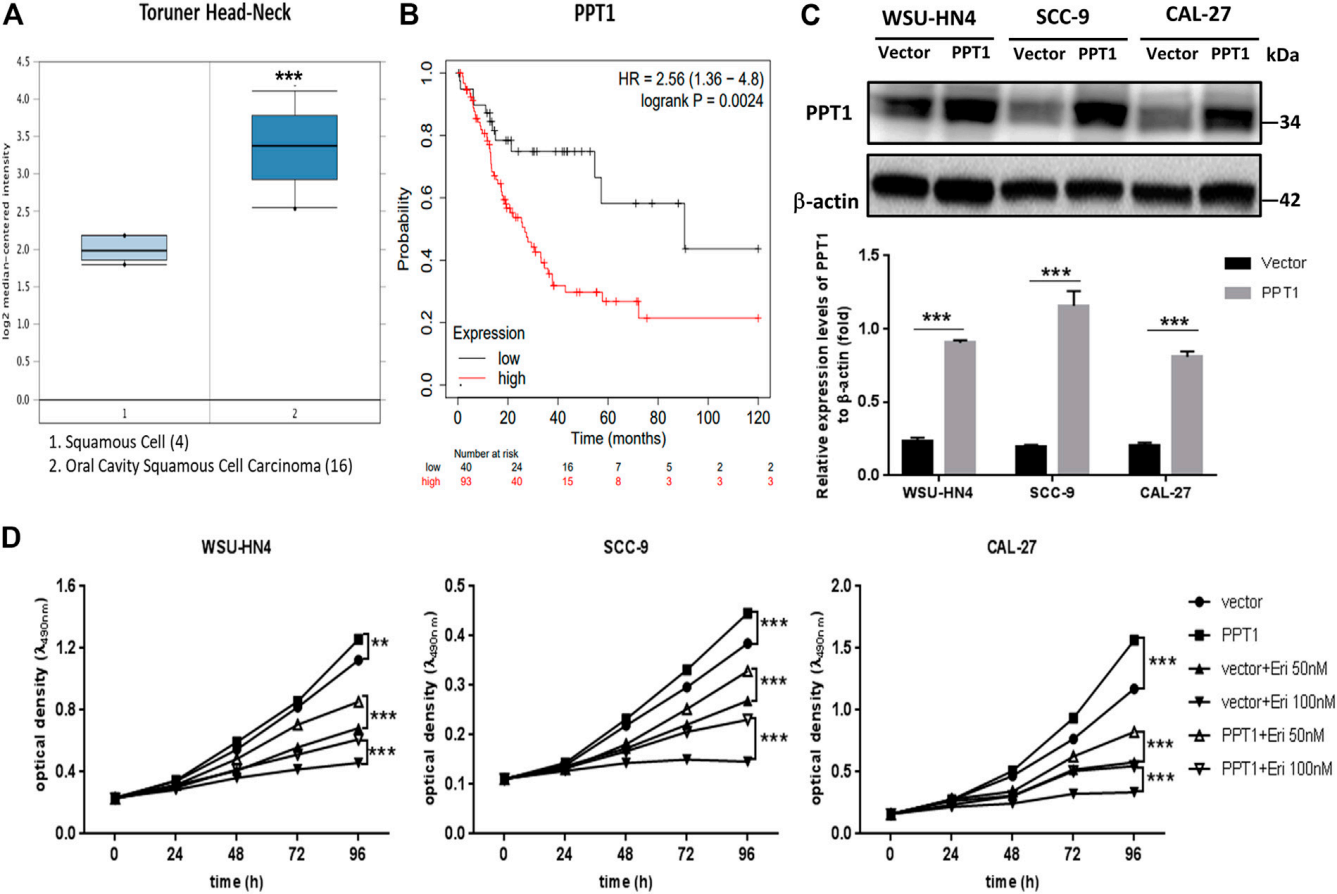
PPT1 reduction contributes to erianin-induced growth inhibition in OSCC cells. **(A)** Analysis of PPT1 gene expression in OSCC (Oncomine database). The box plots were adopted from the gene expression data in Oncomine, which compared PPT1 gene expression in normal squamous cells (left plot) and OSCC cells (right plot). **(B)** Kaplan-Meier analysis of OSCC patients with different PPT1 expression levels. **(C)** Lentivirus particles were transfected into OSCC cells, and PPT1 expression was detected by western blot. **(D)** OSCC cells with vector transfection or PPT1 overexpression were seeded and treated with erianin. Cell viabilities were detected by the MTT assay. (**p < 0.01, ***p < 0.001).

PPT1 has been reported to be overexpressed in cancer and is considered a valid target to control tumor growth (Rebecca et al., 2019; Sharma et al., 2020). To identify whether erianin-induced PPT1 reduction contributed to reduced OSCC cell viability, we first analyzed the expression and potential function of PPT1 in OSCC using online databases. Higher expression levels of PPT1 were found in OSCC epithelia than in normal squamous epithelia, with a fold change of 2.577 and a *p*-value of 2.14E-7 (Oncomine database, Toruner Head-Neck, Figure 7A). Moreover, Kaplan-Meier survival analysis demonstrated that female OSCC patients with higher PPT1 expression had a worse prognosis (hazard ratio = 2.56, *p* = 0.0024, Figure 7B), suggesting that PPT1 promoted OSCC progression and may be a good target in OSCC therapy. We next overexpressed PPT1 in OSCC cells with lentivirus particle transfection. The transfection efficiency was confirmed by western blot, as shown in Figure 7C. OSCC cells transfected with vector or PPT1 were later treated with erianin and cell viabilities were determined by MTT assay. The results showed that PPT1 overexpression significantly reversed erianin-induced inhibition of cell viability (Figure 7D). Hence, these data indicated that PPT1 reduction contributed to erianin-induced growth inhibition in OSCC cells.

### Erianin Inhibited the Growth of OSCC Cells *In Vivo*

**FIGURE 8:**
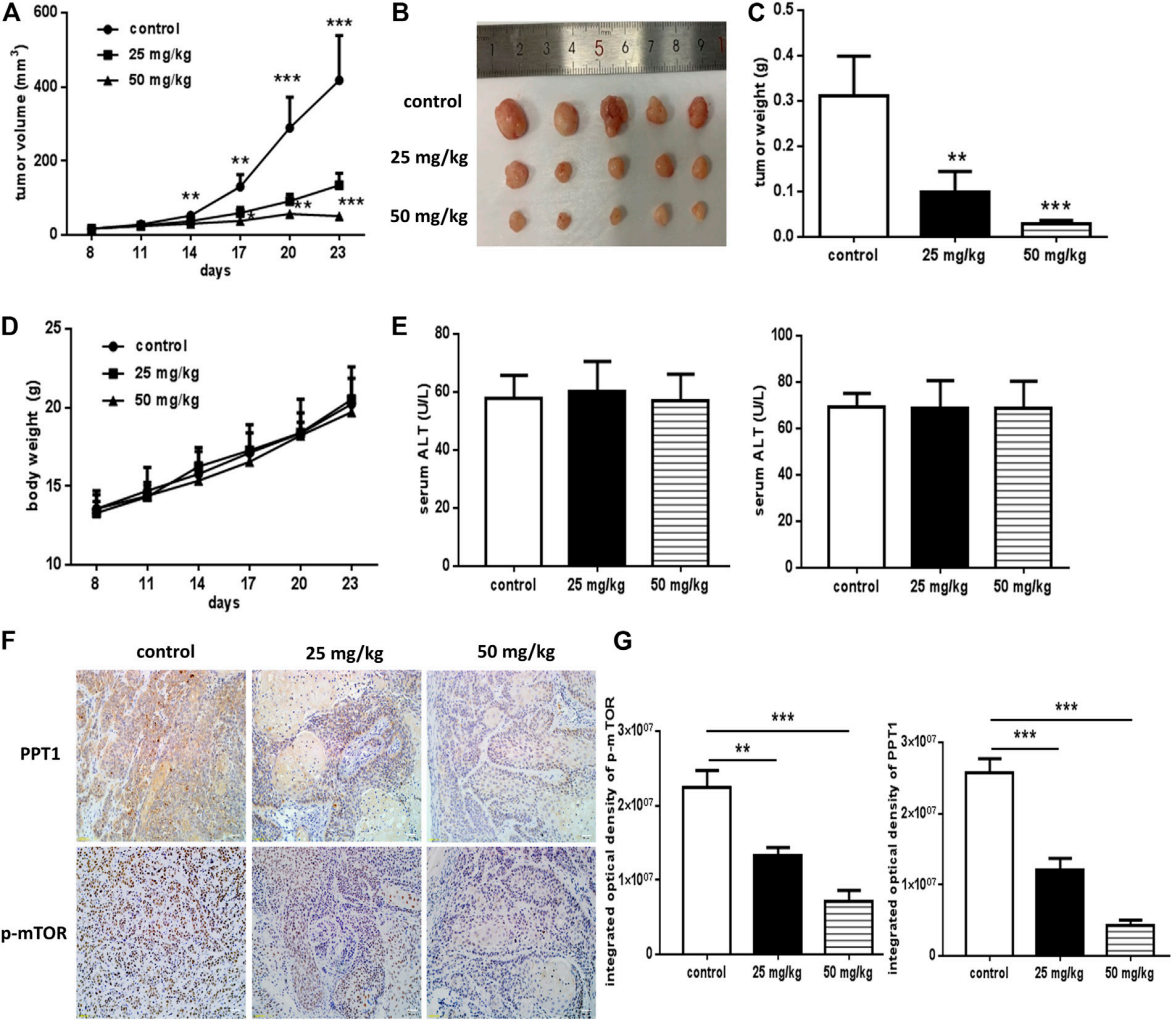
*In vivo* anti-OSCC effects of erianin. The xenograft model was established, and mice were grouped and treated as described previously. **(A)** Graphs representing the average tumor volumes of the xenografts. **(B)** Representative images of the xenografts. **(C)** Weights of the tumors obtained from mice treated with or without erianin. **(D)** Body weights of the mice were recorded. **(E)** Serum aminotransferases levels were measured. **(F)** PPT1 and p-mTOR expression in the xenografts. **(G)** Integrated optical density values of the IHC staining. (Scale bars, 100 μm) Five mice were included for each group, and one representative experiment out of three is shown. (*p < 0.05, ***p < 0.001).

To investigate the therapeutic potential of erianin in OSCC *in vivo*, the xenograft model was established. The animals were inoculated, grouped and treated as described in Materials and Methods Tumor sizes were measured every 3 days and volumes were calculated. Body weight of the mice was recorded. After sacrificed, the tumors were separated and weighed. As shown in Figures 8A–C, erianin treatment significantly reduced the tumor volume and weight. No obvious differences were found in the body weight or serum aminotransferases levels of mice from the three groups (Figures 8D,E). Also, there was no visible differences in the appearance or movement of the mice. To identify the *in vivo* effects of erianin on the expression of PPT1 and p-mTOR, IHC staining of tumor sections from different groups were performed. Markedly, the expression of PPT1 and p-mTOR decreased in tumor cells derived from erianin-treated mice, as shown by the reduced brown staining (Figures 8F,G). So, erianin showed potentially therapeutic effect of OSCC *in vivo*.

## Discussion

The present study aimed to explore the anticancer efficacy of erianin in human OSCC cells and elucidate the related mechanisms. Results indicated that erianin could significantly inhibit OSCC cell growth both *in vitro* and *in vivo*. Erianin induced arrest of the cell cycle at the G2/M phase, induction of apoptosis and pyroptosis, and impairment of autophagic flux in OSCC cells. Further study demonstrated that PPT1 reduction contributed to erianin-induced growth inhibition in OSCC cells. In a xenograft mouse model of OSCC, we confirmed the growth inhibitory effects of erianin *in vivo*, as well as the inhibition of PPT1 and p-mTOR.

The antineoplastic effects of erianin have been observed in a variety of tumors, including osteosarcoma (Wang et al., 2016), hepatic cancer (Dong et al., 2020), lung cancer (Chen P. et al., 2020) and cervical cancer (Yang et al., 2021). In these studies, erianin was demonstrated to modulate the ROS/JNK signaling pathway thus promote G2/M phase blockage, apoptosis, and autophagy (Wang et al., 2016), or induce Ca^2+^/CaM-dependent ferroptosis and inhibite cell migration (Chen P. et al., 2020), or inhibit PD-L1 expression and enhance cytotoxic T lymphocyte activity (Yang et al., 2021). Our results indicated that erianin also induced G2/M arrest and apoptosis in OSCC cells. Moreover, we found that erianin induced GSDME-mediated pyroptosis and impaired autophagic flux in OSCC cells. Therefore, erianin has a comprehensive antitumor effect, but the related mechanisms may differ depending on the cancer cell type. Exploring potentially new mechanisms may help to improve the efficacy and reduce side effects during its therapeutic application.

Recently, a new form of programmed cell death (PCD) named pyroptosis has been identified, which is characterized by the release of inflammatory cytokines and cell swelling with bubbles. The gasdermin family which share a pore-forming domain is considered the key factor mediating pyroptosis (Hachim et al., 2020; Liu et al., 2021). Classic pyroptosis usually refers to gasdermin D (GSDMD)-mediated pyroptosis, in which full-length GSDMD was cleaved into a C-terminal fragment and an N-terminal fragment by activated caspase 1/4/5/11. The N-terminus finally translocates to the membrane to form the pore, leading to cell death. Yuan *et al.* reported that cucurbitacin B (an extract from muskmelon pedicels) could directly bind to Toll-like receptor 4 (TLR4) and activate the NLRP3 inflammasome, which further promoted the production of pore-forming N-terminals of GSDMD to execute pyroptosis in non-small cell lung cancer (Yuan et al., 2021). Moreover, nonclassical or secondary pyroptosis induced by caspase 3 cleavage of GSDME has been observed in cancer cells treated with chemotherapy drugs such as lobaplatin and navitoclax (Hu et al., 2020). Thus, we further investigated the possible involvement of pyroptosis in erianin-treated OSCC cells. There was no obvious change of GSDMD, while the N-terminal of GSDME was significantly increased in OSCC cells with erianin treatment. Moreover, the release of LDH and IL-1β was also strikingly elevated in the culture supernatant, indicating that erianin destroyed the integrity of OSCC cell membrane. Hence, GSDME-mediated pyroptosis was reported for the first time to be involved in erianin-induced cell death *in vitro*.

Autophagy is an importantly conserved process existing in almost all kinds of cells at low basal levels, but is upregulated when cells face therapeutic or metabolic stress (White et al., 2021). By degrading intracellular damaged organelles and captured proteins in lysosomes, autophagy reuses the degradation products to sustain survival. In many cancers, autophagy is increased to facilitate the rapid growth of the tumor cells (Xia et al., 2021; Zhang and Liu, 2021). However, excessive autophagy leads to nonapoptotic programmed cell death. Thus, targeting autophagy is another therapeutic strategy in cancer therapy. Man *et al.* found that W436 induced protective autophagy in hepatocellular carcinoma cells by inhibiting the protein kinase B (PKB)/mTOR pathway (Man et al., 2021). Coronel-Hernández *et al.* reported that the combined use of sodium oxamate, metformin and doxorubicin in colorectal cancer cells induced death-promoting autophagy by downregulating hypoxia-inducible factor (HIF)-1α (Coronel-Hernandez et al., 2021). Previous studies indicated that erianin also induced autophagy in cancer cells (Wang et al., 2016; Chen Y. T. et al., 2020). In our study, we found increased levels of LC3-II and beclin-1 in OSCC cells 24 h after erianin treatment, similar expression patterns to OSCC cells treated with the late phage autophagy inhibitor CQ. This result implied that erianin induced the formation of autophagosomes but impaired the autophagic flux. P62 plays a key role in regulating autophagy via binding to the protein aggregates and leading them into autolysosomes for further degradation. Classically, the p62 level is expected to decrease during autophagy due to degradation. However, p62 levels in OSCC cells increased after erianin treatment for 24 h, indicating potentially affected lysosomal function. The use of mCherry-GFP-LC3 and mCherry-p62 adenovirus plasmids which monitors the autophagosomes and autolysosomes formation further confirmed the western blotting results. The increased yellow puncta of mCherry-GFP-LC3 and increased size and number of mCherry-p62 in erianin-treated OSCC cells intuitively demonstrated impaired autophagic flux.

We then went further to determine the inner mechanisms. Recently, PPT1 has received much attention as a new regulator of autophagy. PPT1 is a lysosomal enzyme which deacylates the palmitoylated proteins. Lack of PPT1 usually leads to a genetic disease named infantile neuronal ceroid lipofuscinosis, which is caused by substantial death of cortical neurons (Koster and Yoshii, 2019). In cancer cells, Amaravadi *et al.* found that PPT1 inhibition disrupted the lysosomal localization of mTOR and inhibited the induction of lysosomal catabolism, resulting in impaired autophagic flux and affected cell growth (Rebecca et al., 2017; Rebecca et al., 2019). To get full activation, mTOR translocates to the lysosomal surface to approach its activator RHEB in an amino acid-dependent manner. The recruitment of mTOR to the lysosome is regulated by the v-ATPase and the Ragulator machinery (Zoncu et al., 2011). Inhibition of PPT1 caused v-ATPase mislocalization from the lysosome and gave rise to two main consequences: 1) the blockade of autophagic flux resulted from lysosomal deacidification and 2) the inhibition of mTOR signaling due to the disrupted physical interaction between v-ATPase and Ragulator (Rebecca et al., 2017; Rebecca et al., 2019). In our study, erianin treatment induced a significant reduction in PPT1 and a decrease in mTOR phosphorylation in OSCC cells. From the PLA assay, the reduced interaction of mTOR and RHEB was observed more intuitively. As PPT1 overexpression significantly reversed the inhibition of cell viability, we speculated that PPT1 reduction played an important role in erianin-induced OSCC cell death.

In conclusion, our study showed that erianin impaired OSCC cell growth both *in vitro* and *in vivo*. The related mechanisms included G2/M phase cycle arrest, apoptosis and pyroptosis induction, and impaired autophagic flux in erianin-treated OSCC cells. Moreover, we identified that PPT1 reduction played a critical role in mediating erianin-induced growth inhibition in OSCC cells. These results show that erianin may have great potential in OSCC therapy.

## Data Availability

The original contributions presented in the study are included in the article/Supplementary material, further inquiries can be directed to the corresponding author.
